# Effects of urban green exercise on mental health: a systematic review and meta-analysis

**DOI:** 10.3389/fpubh.2025.1677223

**Published:** 2025-09-25

**Authors:** Guidan Hu, Qingyuan Luo, Peng Zhang, Hao Zeng, Xiujie Ma

**Affiliations:** ^1^School of Wushu, Chengdu Sport University, Chengdu, China; ^2^College of Physical Education and Sports, Beijing Normal University, Beijing, China; ^3^School of Wushu, Tianjin University of Sport, Tianjin, China; ^4^Chinese GuoShu Academy, Chengdu Sports University, Chengdu, China

**Keywords:** urban green exercise, mental health, systematic review, meta-analysis, randomized controlled trial, green space, public health

## Abstract

As global urbanization accelerates, concerns regarding the mental health of urban residents have become increasingly prominent. Urban green exercise, a non-pharmacological intervention integrating exposure to nature with physical activity, has gained considerable attention due to its potential mental health benefits. However, systematic evidence synthesizing the specific effects and underlying mechanisms of urban green exercise on mental health remains limited. Following strict adherence to PRISMA guidelines, systematic searches of PubMed, MEDLINE, Embase, the Cochrane Library, and Web Of Science identified 15 RCTs involving urban green spaces, comprising 980 participants aged 18 years and older. Methodological quality was evaluated using the Cochrane RoB 2.0 tool. Meta-analysis using standardized mean difference (SMD) was conducted using Stata 17.0, while subgroup and regression analyses were performed to explore moderating factors, including intervention period, frequency, duration per session, exercise intensity (METs), and gender. A moderate and statistically significant positive impact of urban green exercise on mental health was found (SMD = −0.40; 95% CI = −0.56 to −0.25; *p* < 0.001), with low between-study heterogeneity (I^2^ = 33.9%). The most pronounced effects were associated with short-duration interventions (<12 weeks), interventions conducted at least three times weekly, session durations of 20 min or less, and low-to-moderate intensity (≤3 METs). Greater benefits were observed among female participants. This systematic review provides robust empirical support for the mental health benefits of urban green exercise in adult urban populations, highlighting the efficacy of short-duration, high-frequency, moderate-intensity intervention models. These findings offer evidence-based insights to inform urban public health policy and green space planning, emphasizing the need to enhance the accessibility and utilization of urban green spaces. Further high-quality RCTs with larger sample sizes are recommended to further validate long-term effects and elucidate underlying mechanisms.

## 1 Introduction

According to the World Health Organization (WHO), nearly 1 billion people wordwide experience mental health conditions of varying degrees, with anxiety and depression being the most common (1). With the accelerating pace of global urbanization, city dwellers have become increasingly susceptible to mood disorders, insomnia, and burnout (2–4), potentially compromising their quality of life and social functioning (5). Currently, mainstream treatments for mental health disorders primarily include pharmacotherapy (6), cognitive behavioral therapy (CBT) (7, 8), and psychological counseling (9). Although these interventions have demonstrated clinical effectiveness, limitations related to accessibility, adherence, and side effects highlight their inadequacy for large-scale, long-term, and low-risk mental health management. Consequently, identifying and promoting more accessible, low-threshold, and sustainable mental health strategies has become a central focus within both academic and public health arenas.

“Green Exercise” refers to physical activity conducted in natural environments, such as grass-coverd areas, vegetated parks, or outdoor sports fields. Green exercise has been recognized not only for its physical health benefits but also significant mental health improvements attributed to natural environmental exposure (10, 11). Considerable preliminary evidence suggests that, compared with indoor or built environments, green exercise is more effective at alleviating symptoms of anxiety and depression (12–14), enhancing mood and self-esteem (15, 16), restoring attention (17–19) and improving overall wellbeing (20). The restorative benefits of nature, as supported by Attention Restoration Theory (21, 22) and Stress Reduction Theory (23, 24), suggest that exercising in natural environments may facilitate recovery from mental fatigue and mitigate both physiological and psychological stress. Although the benefits of “green exercise” have gained initial recognition, most foundational research has concentrated on pristine, non-urban environments such as rural areas and forests, which are not readily accessible to the majority of urban dwellers. Consequently, urban green exercise conducted in settings such as city parks and community greenways, holds greater relevance for the daily routines of contemporary urban populations. Its intervention effects and practical value therefore merit rigorous scientific investigation. To date, findings from research on urban green exercise have been inconclusive. For instance, while some studies have confirmed significant mental health benefits among middle-aged and older adults (25, 26), others targeting children and adolescents did not demonstrate a definitive benefit over indoor alternatives (27). Furthermore, intervention effects have been shown to be influenced by factors such as environmental type (28, 29) and intervention duration (30). These discrepancies across populations (e.g., older adults vs. adolescents), environments (e.g., forests vs. urban parks), and intervention durations indicate that inconsistencies in findings may be attributable to methodological heterogeneity among studies.

In order to address these inconsistencies and generate more robust evidence for practical application, a systematic review and meta-analysis of randomized controlled trials (RCTs) on urban green exercise will be conducted. The moderating effects of key variables—including exercise intensity, intervention duration, frequency, and session length—on mental health outcomes will be systematically examined. This study aims to propose an evidence-based intervention protocol for urban green exercise, thus offering a theoretical foundation and a practical pathway for promoting urban mental health and informing public health policy.

## 2 Materials and methods

### 2.1 Search strategy

To ensure transparency and reproducibility, this systematic review was conducted and reported in accordance with the Preferred Reporting Items for Systematic Reviews and Meta-Analyses (PRISMA) guidelines (31). The review protocol was registered in the PROSPERO database (CRD420251115871). All steps outlined in the PRISMA statement were meticulously followed throughout the review process. A comprehensive literature search was conducted in PubMed, Embase, MEDLINE, the Cochrane Library, and Web of Science for all relevant literature published up to June 20, 2025, without language restrictions.

### 2.2 Inclusion and exclusion criteria

The inclusion criteria were as follows: (1) RCTs explicitly evaluating the effects of green exercise interventions on mental health outcomes; (2) participants aged 18 years or older; (3) interventions conducted in urban natural environments, including city parks, neighborhood green areas, urban forests, or campus green spaces, combining nature exposure and physical activities such as walking, cycling, aerobics, or running; (4) a clearly defined control group was required, such as a waitlist control, usual activity group, indoor exercise group, or other non-green exercise intervention group; (5) at least one mental health outcome (e.g., depression, anxiety, stress, self-esteem, wellbeing, or quality of life) was required to be reported using quantitative tools; (6) complete pre-intervention and post-intervention data, or sufficient statistical information to calculate effect sizes (e.g., means, standard deviations, sample sizes), was required. Studies were excluded if they: (1) employed non-RCTs designs (e.g., observational, cross-sectional, qualitative studies, or reviews); (2) involved only static nature exposure (e.g., sitting meditation or viewing nature images); (3) were conducted in non-urban settings such as forests, rural, or coastal regions; (4) lacked reported mental health outcomes or extractable data; or (5) assessed only the frequency of green space exposure without an intervention component. Additionally, conference abstracts, dissertations, unpublished preprints, and non-peer-reviewed articles were excluded. Titles and abstracts were independently screened according to inclusion and exclusion criteria, and duplicates were removed. Disagreements were resolved through discussion, with a third reviewer consulted when consensus could not be reached.

### 2.3 Data extraction

Literature screening, data extraction, and cross-checking were independently conducted by two reviewers. When outcome data were reported graphically or post-intervention results were incomplete, corresponding authors were contacted for numerical data. Extracted data included: (1) general study information (e.g., first author, year of publication, country of study, sample size); (2) baseline participant characteristics (e.g., age, sex); (3) intervention protocols (e.g., type of intervention, total duration, weekly frequency, and duration per session); (4) instruments used for psychological health assessment; and (5) outcome measures. Any disagreements during the extraction process were resolved by a third reviewer.

### 2.4 Quality assessment

Data related to participants, interventions, and outcomes were independently extracted by two reviewers using a standardized extraction form. Concurrently, the risk of bias in all included randomized controlled trials was systematically assessed using the Cochrane Risk of Bias tool 2 (RoB 2) (32). Five bias domains were evaluated: (1) bias arising from the randomization process; (2) bias due to deviations from intended interventions; (3) bias due to missing outcome data; (4) bias in measurement of the outcome; and (5) bias in selection of the reported result. Each domain, as well as the overall risk of bias, was classified as “low risk,” “some concerns,” or “high risk” according to the criteria specified in the Cochrane Handbook for Systematic Reviews of Interventions. Any disagreements during the assessment process were resolved through discussion. If consensus could not be reached, a third reviewer was consulted for the final determination.

### 2.5 Statistical analysis

The methodological quality of included studies was assessed using the Cochrane Risk of Bias tool (RoB 2). Statistical analyses—including data synthesis, heterogeneity testing, forest plot generation, and publication bias evaluation—were conducted using Stata 17.0. Because most mental health outcomes in the included studies were continuous variables measured using different instruments and units, the standardized mean difference (SMD) with 95% confidence intervals (95% CI) was used as the summary effect size. The SMD was computed as the difference between the mean values of the intervention and control groups, divided by the pooled standard deviation, incorporating Hedges’ g correction for small sample sizes when appropriate. Post-intervention means and standard deviations were prioritized; when only change scores were available, standardized mean differences based on change scores were computed and included in the same analytical model. Between-study heterogeneity was assessed through Cochran’s Q test and quantified by the I^2^ statistic. A significant Q test is indicative of heterogeneity, whereas I^2^ represents its magnitude; values of approximately 25, 50, and 75% are conventionally interpreted as low, moderate, and high heterogeneity, respectively (33, 34). Although an I^2^ value below the 50% threshold is commonly considered justification for a fixed-effect model, the use of a random-effects model was prespecified to synthesize the results. This was considered a more conservative approach, allowing for potential true variability in populations, interventions, and outcome measurements across studies. Publication bias was assessed using Begg’s rank correlation and Egger’s regression tests, complemented by visual inspection of funnel plots. Sensitivity analyses were performed to evaluate the robustness of results and identify potential influential studies affecting the pooled effect size.

## 3 Results

### 3.1 Studies selection

**Figure 1:**
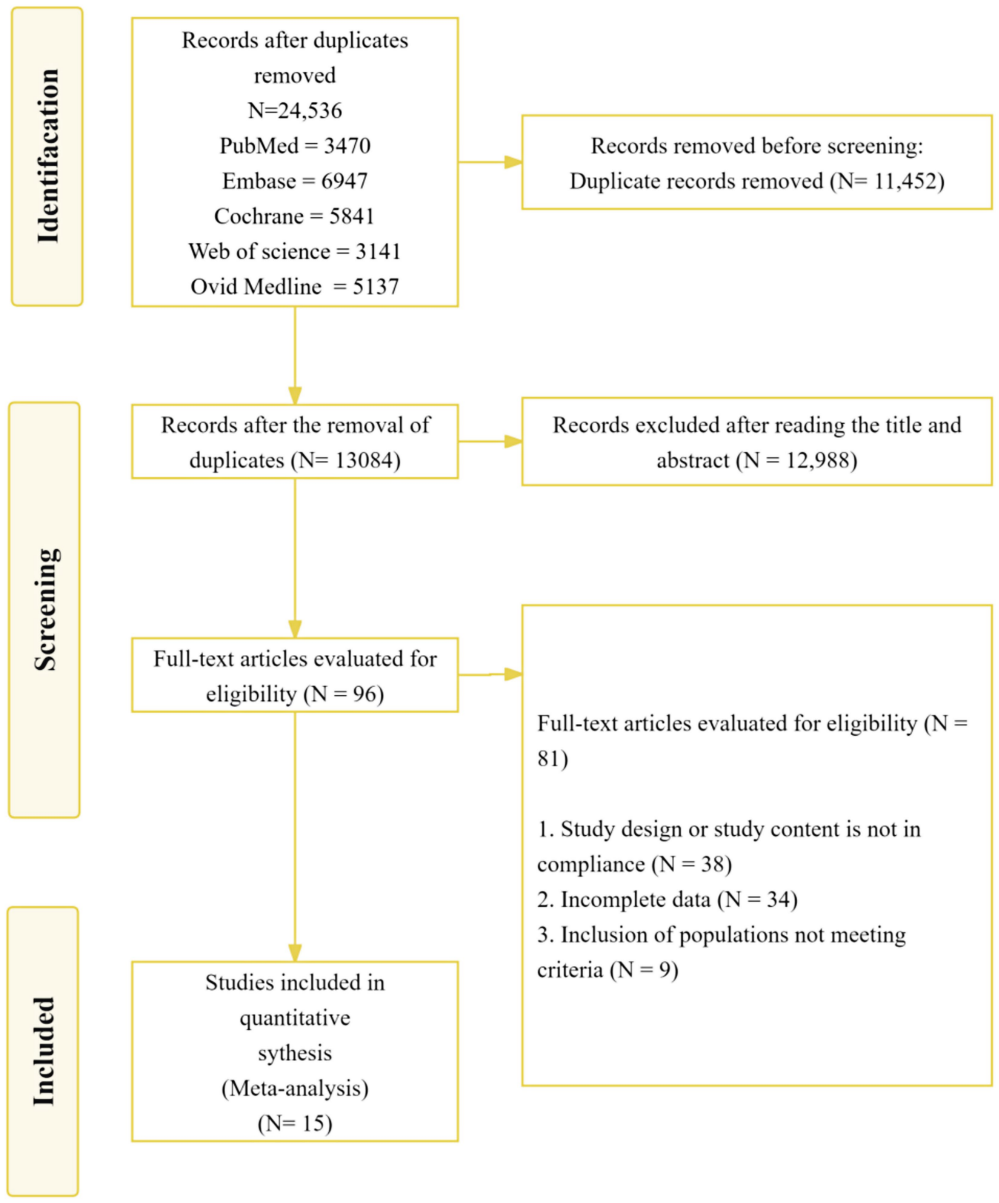
Flow of study selection.

A comprehensive literature search of PubMed, MEDLINE, Embase, the Cochrane Library, and Web of Science was performed up to June 20, 2025, identifying a total of 24,536 records. During the initial screening, 11,452 duplicate records and 12,988 irrelevant studies were excluded after reviewing titles and abstracts. Ninety-six studies underwent full-text review for eligibility, of which 82 were excluded due to the following reasons: inappropriate intervention or content (*n* = 38), incomplete outcome data (*n* = 34), and ineligible population (*n* = 9). Ultimately, as illustrated in the PRISMA flow diagram (Figure 1), 15 studies met the inclusion criteria and were included in the final meta-analysis. Table 1 summarizes the characteristics of these included studies.

**Table 1:** Included trial characteristics.

Trial ID	Time	Country	N	Age mean (range)	Gender, % Female	T	C	Outcome measures	α
Dickmeyer et al.	2025	Australia	37	18–70	0	20	17	DASS-21 MDRS-2WEMWBS	DASS-21 = 0.91 MDRS-22:0.8 WEMWBS:0.77
Watkins-Martin et al.	2022	Canada	37	18–65	67.57	20	17	PANAS HAM-D	PANAS = 0.88–0.93 HAM-D = 0.89–0.93
Bang et al.	2016	South Korea	45	30–53	93.3	18	27	HPLP IIK-BDI GHQ/QL-12	HPLP II = 0.92; K-BDI = 0.76 GHQ/QL-12 = 0.83
Brito et al.	2024	Portuga	104	18–50	31.73	51	53	FS RPE	NR
Rantanen et al.	2015	Finland	121	>65	90.08	60	61	WHOQOL-BREF	WHOQOL-BREF = 0.846
Vert et al.	2020	Spain	59	18–65	69.49	59	59	WHO-5 TMD SF-36	NR
Hvid et al.	2025	Denmark	62	27–68	79.03	33	29	WHO5 HR-QoL VAS	NR
Lee et al.	2025	Hong Kong, China	34	≥55	70.59	16	18	SWEMWBS	NR
Ryu et al.	2020	South Korea	60	18–65	46.67	30	30	BDI STAI BPRS WHOQOL-BREF	BDI = 0.84 STAI=NR BPRS=NR WHOQOL-BREF = 0.89
Levinger et al.	2023	Australia	16	≥60	87.5	8	8	GDS-15 UCLA 3	NR
Park et al.	2024	South Korea	63	>18	80.95	36	27	K-POMS-B	NR
Müller-Riemenschneider et al.	2020	Singapore	160	40–65	95.49	80	80	SF-12 WHO5 WHOQOL-BREF	NR
Rogerson et al.	2020	The United Kingdom	40	21–68	50	40	40	POMS	NR
Brown et al.	2014	The United Kingdom	94	18–65	21.28	65	29	SF - 8	NR
Sales et al.	2017	Australia	48	60–90	43.75	27	21	SF-12	NR

DASS-21, Depression Anxiety Stress Scales-21; MDRS-2, Montgomery–Asberg Depression Rating Scale, Second Edition; WEMWBS, Warwick-Edinburgh Mental Wellbeing Scale; PANAS, Positive and Negative Affect Schedule; HAM-D, Hamilton Depression Rating Scale; HPLP II, Health-Promoting Lifestyle Profile II; K-BDI, Korean version of the Beck Depression Inventory; GHQ-12, General Health Questionnaire-12; FS, Flourishing Scale; RPE, Rating of Perceived Exertion Scale; WHOQOL-BREF, World Health Organization Quality of Life-BREF; WHO-5, World Health Organization-Five Well-Being Index; TMD, Total Mood Disturbance; SF-36, Short Form Health Survey-36; HR-QoL VAS, Health-Related Quality of Life Visual Analog Scale; SWEMWBS, Short Warwick–Edinburgh Mental Wellbeing Scale; BDI, Beck Depression Inventory; STAI, State–Trait Anxiety Inventory; BPRS, Brief Psychiatric Rating Scale; GDS-15, Geriatric Depression Scale-15 items; UCLA 3, UCLA Loneliness Scale, Version 3; K-POMS-B, Korean version of the Profile of Mood States-Brief; SF-12, Short Form Health Survey-12.

### 3.2 Characteristics of eligible studies

A total of 15 RCTs published across various countries were included in this systematic review: Australia (35–37), Canada (38), South Korea (39–41), Portugal (42), Finland (43), Spain (44), Denmark (45), Singapore (46), the United Kingdom (47, 48), and Hong Kong, China (49). Sample sizes ranged from 16 to 160 participants, comprising a total of 980 adults aged 18 years or older. All studies employed an RCT design, with follow-up durations ranging from 1 to 5 months.

### 3.3 Interventions and controls

Characteristics of the urban green exercise interventions from all included studies are presented in Table 2. All interventions were conducted in urban natural environments, primarily urban green spaces such as city parks, parklands, and urban forests, with one study conducted on a campus. Walking interventions were utilized in seven studies; aerobic exercises in three; cycling in three; and yoga or movement-based activities in one. Intervention intensity was standardized by converting activity types into metabolic equivalents (METs) based on international physical activity classifications. Specifically, four studies involved light-intensity activities (≤3 METs) (37, 38, 45, 50), six studies included moderate-intensity activities (3 to <6 METs) (39–41, 43, 46, 47), and five studies comprised vigorous-intensity activities (≥6 METs) (42, 44, 48, 49, 51). Waitlist control (WLC) groups were employed in six studies (41, 45–48, 51), while others utilized controls such as usual care, individual running, or cognitive behavioral therapy/educational programs. Active controls (e.g., occupational therapy, physical fitness training) were also used to ensure structural equivalence and enhance comparative validity. Intervention periods ranged from one to 36 weeks, with frequencies varying between one and six sessions per week. Session durations ranged from 12 to 120 min, typically lasting between 30 and 90 min. Most interventions featured structured schedules supervised by on-site personnel, such as exercise instructors, health coaches, or project leaders. Overall, the typical intervention protocol involved moderate-intensity exercise conducted once or twice weekly, lasting 30–90 min per session.

**Table 2:** Characteristics of included trials.

Trial ID	Intervention content	Intervention site	Setting	Duration	Session (Min)	Sessions per Week	Control Arm	MET
Dickmeyer et al.	1)Walking	Campus green space	S I NR	6 weeks	60	1	Usual indoor therapy	2.5
Watkins-Martin et al.	1)Cycling	Park green space	S Q G	1 weeks	60	1	Cycling in gray space	5
Bang et al.	1)Walking	Urban forest	S Q G	10 weeks	40	2	WLC	3.5
Brito et al.	1)Aerobic training	Urban park	S Q G	1 weeks	8	1	Indoor aerobic training	8
Rantanen et al.	1)Walking	Urban park	S I G	12 weeks	20	1	WLC	2.5
Vert et al.	1)Walking	Urban park	U Q G	12 weeks	20	4	WLC	3.5
Hvid et al.	1)Walking	Urban park	S Q G	14 weeks	12–40	2	WLC	5
Lee et al.	1)Walking	Urban green space	S Q G	4 weeks	60	1	Educational program (e.g., chair yoga, seated Baduanjin, etc.)	2.5
Ryu et al.	1)Cycling	Park green space	S Q G	16 weeks	90	1	Occupational therapy	7
Levinger et al.	1)Exercise	Park green space	S Q G	24 weeks	60–90	2	Usual care program (facility-organized recreational and leisure group activities)	3
Park et al.	1)Yoga, movement games	Urban forest	S Q G	8 weeks	120	2	Educational program	2.5
Müller-Riemenschneider et al.	1)Group exercise	Urban park	S Q G	26 weeks	60	1	WLC	8
Rogerson et al.	1)Team running	Urban park	S Q NR	2 weeks	10–20	1	Individual running	8
Brown et al.	1)Walking	Urban green space	U I NR	16 weeks	20	2	WLC	7
Sales et al.	1)Exercise	Urban park	S Q G	36 weeks	60–90	2	Physical fitness training	3.5

WLC, waitlist control; NR, no report; S, supervised; Q, group; U, unsupervised; I, individual; NR, not reported or unclear.

### 3.4 Risk of bias

**Figure 2:**
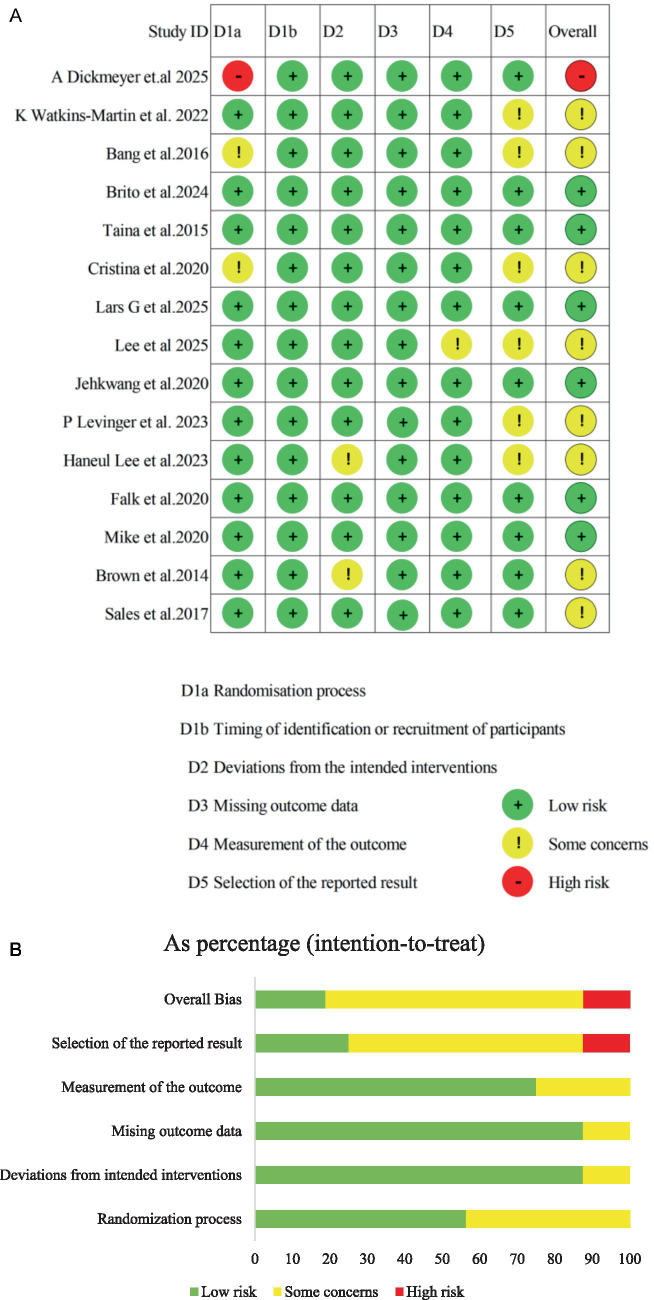
**(A)** Risk of bias ratings. **(B)** Risk of bias graph.

The methodological quality of the 15 RCTs included in the meta-analysis was systematically assessed using the RoB 2. A total of six studies (40, 42, 43, 45, 46, 48) were rated as “low risk” of bias across all domains. Eight studies (36–39, 41, 44, 47, 49) were rated as having “some concerns,” and one study (35) was rated as “high risk” due to serious deficiencies in the randomization process and selection of the reported result (see Figures 2A,B for details). Specifically, in the domain of bias arising from the randomization process (D1), three studies (35, 39, 44) were rated as having “some concerns” or “high risk” due to unclear reporting of random sequence generation or potential selection bias, whereas the remaining studies were rated as low risk. For the timing of recruitment, a low risk of bias was observed in all studies. The overall risk of bias due to deviations from intended interventions (D2) was considered low, with 13 studies (35–40, 42–46, 48, 49) rated as low risk and two (42, 47) as having “some concerns,” indicating that most studies maintained high fidelity to their original intervention protocols. In the domain of bias due to missing outcome data (D3), all studies were rated as low risk, as completeness of outcome data was clearly reported, thereby precluding bias from sample attrition. For bias in measurement of the outcome (D4), 14 studies (35–48) were rated as “low risk” due to the use of consistent, validated scales and standardized assessment procedures, while one study (47) was rated as having “some concerns” owing to ambiguity in assessment tools or unclear reporting of scoring procedures. The highest risk of bias was observed in the domain of selection of the reported result (D5). Low risk was assigned to nine studies (35, 37, 40, 42, 43, 45–48) because a complete study protocol or a pre-specified statistical analysis plan was registered on a trial registry. The remaining six studies (36, 38, 39, 41, 44, 49) were rated as having “some concerns” due to insufficient transparency in their analysis plan or failure to specify reporting standards. Overall, a risk of bias in at least one domain was observed in approximately 40% of the included studies, primarily in the areas of randomization and selection of reported results. The internal validity and interpretability of the meta-analytic findings may therefore be limited.

### 3.5 Overall intervention effect

#### 3.5.1 Exercise group and control group

**Figure 3:**
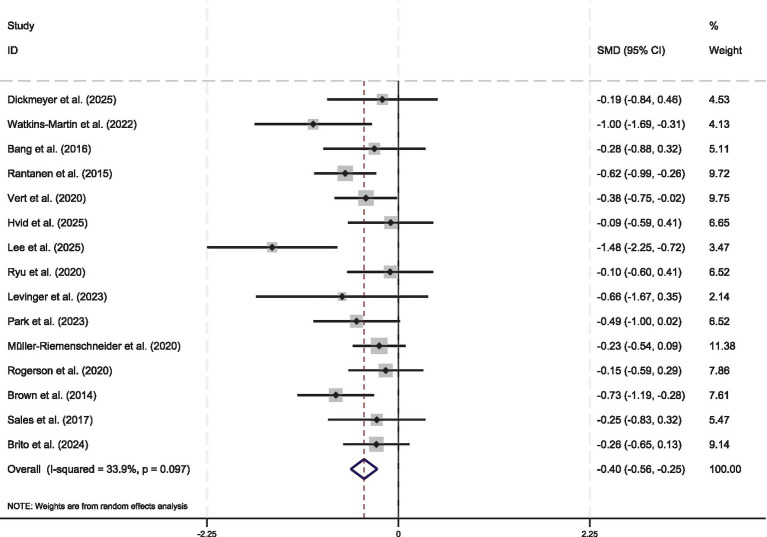
Standardized effect sizes of individual studies on urban green exercise interventions for mental health. The figure depicts the effect size from each of the 15 individual studies as gray squares, with the square area proportional to the corresponding study’s weight in the meta-analysis. Horizontal lines extending from each square indicate the 95% CI for the estimated effect size of each study. The diamond at the bottom denotes the pooled effect size derived from the fixed-effect model, with its width denoting the 95% CI. The vertical dashed line at zero denotes the no-effect point. Negative effect sizes (i.e., those to the left of zero) indicate an effect favoring the urban green exercise intervention. The I^2^ statistic quantifies the proportion of total variation across studies attributable to between-study heterogeneity. SMD, standardized mean difference; CI, confidence interval.

This meta-analysis comprised 15 studies that assessed the effects of urban greenspace exercise interventions on mental health outcomes. Low to moderate between-study heterogeneity was observed (I^2^ = 33.9%, Q = 20.32, *p* = 0.097). Although an I^2^ value below the conventional 50% threshold often justifies the use of a fixed-effect model, a random-effects model was prespecified to account for potential true variability attributable to differences in participant demographics, intervention modalities, duration, intensity, and outcome measurements, even in the presence of modest statistical heterogeneity. Effect sizes were reported as SMD, calculated as the difference between intervention and control group means divided by the pooled standard deviation; negative SMD values reflected greater improvement in symptom-related outcomes for the intervention group. The pooled estimate yielded a statistically significant moderate effect favoring urban greenspace exercise (SMD = −0.40; 95% CI = −0.55 to −0.24; *p* < 0.001). These findings indicate that urban greenspace exercise interventions are associated with significantly greater reductions in symptom-related outcomes compared to control conditions, such as no intervention or non-green/indoor comparators. It should be noted that the effect sizes of certain individual studies were close to null or had confidence intervals including zero. This may be attributable to factors such as insufficient intervention durations or small sample sizes in those studies (Figure 3).

#### 3.5.2 Regression analysis

To examine the linear relationship between intervention parameters and effect size, a multivariable meta-regression analysis was performed. In this model, the SMD from each study was used as the dependent variable. Several pre-specified covariates were simultaneously included in the model as potential moderators (independent variables): intervention duration (weeks), METs, session length (minutes), intervention type, and gender composition. It has been suggested in previous literature that different intervention characteristics may affect mental health outcomes to varying degrees by modulating physiological arousal and emotional regulation mechanisms (50). Among these, METs have been used to quantify the physiological load and stimulation associated with various physical activities (51). Accordingly, METs were included as an intensity indicator in the subgroup regression analyses to explore their potential contribution to the heterogeneity of effect sizes. As shown in Table 3, no statistically significant impact of these covariates on effect size was observed (all *p* > 0.05). Specifically, intervention duration (*β* = 0.059, 95% CI = −0.246–0.364, *p* = 0.674) was found to have a very weak predictive effect on effect size; intervention frequency (*β* = 0.34, 95% CI = 0.307–0.375, *p* = 0.826) was negatively related but not significant; and session length (*β* = 0.007, 95% CI = −0.277–0.292, *p* = 0.953), intervention intensity (METs) (*β* = 0.174, 95% CI = −0.133–0.480, *p* = 0.232), and gender composition (*β* = −0.052, 95% CI = −0.544–0.439, *p* = 0.815) also demonstrated no significant effects. These results suggest that the basic intervention parameters or sample characteristics included in the model were unable to account for the observed differences in effect sizes across studies, indicating that other unmeasured moderating factors—such as psychological engagement, environmental context, or baseline mental health status—may have contributed. These findings indicate that, although no statistically significant linear trend was observed, the potential presence of nonlinearity or threshold effects cannot be ruled out. In accordance with the prespecified analysis plan, subgroup analyses were conducted to identify practice-relevant “optimal dose” ranges for exercise prescription.

**Table 3:** Multivariate meta-regression analysis of mental health.

ES	Coefficient	Std. err.	*t*	*P* > |t|	[95% conf. interval]
Duration (weeks)	0.0587264	0.1350321	0.43	0.674	−0.2467375–0.3641904
Frequency (week)	0.340603	0.1508192	0.23	0.826	−0.3071164–0.3752371
Time (min)	0.0075852	0.1258165	0.06	0.953	−0.2770315–0.2922019
METs	0.1738496	0.1357357	1.28	0.232	−0.1332059–0.480905
Gender	−0.052492	0.2173498	−0.24	0.815	−0.5441714–0.4391873
_cons	−0.8755861	0.6637296	−1.32	0.220	−2.377047–0.6258744

ES, effect size (used in original table header, clarified as SMD); Std. err., standard error; CI, confidence interval; METs, metabolic equivalents.

#### 3.5.3 Sub-group analysis

With the goal of informing an urban green exercise prescription, prespecified subgroup analyses were conducted—using data from all included trials—based on intervention duration (weeks), weekly frequency, session duration (minutes), exercise intensity (METs), and gender, in order to identify practice-relevant optimal dose ranges in urban settings (see Table 4). All subgroup analyses were performed using a random-effects model, with pooled effect sizes (SMD), confidence intervals, and heterogeneity statistics reported. The results of the subgroup analyses were as follows: The most significant improvements in mental health were observed for short-term interventions (<12 weeks; SMD = −0.481, 95% CI = −0.788 to −0.173, *p* = 0.002, I^2^ = 54.0%). A notable effect was observed for medium-term interventions (12 to <16 weeks; SMD = −0.414) with lower heterogeneity (I^2^ = 37.0%). A comparatively smaller effect was found for long-term interventions (≥16 weeks; SMD = −0.261), This result may seem counterintuitive when compared with the assumption that continuous, long-term exercise confers the greatest benefits; potential mechanisms underlying this pattern are examined in the Discussion section. Significant improvements in mental health outcomes were found across interventions conducted once per week (SMD = −0.428), twice per week (SMD = −0.412), or three or more times per week (SMD = −0.382), with all *p* ≤ 0.040 and negligible heterogeneity (I^2^ = 0.0%), indicating consistent efficacy across frequency levels. The largest effect size was observed in the subgroup with interventions lasting less than 20 min per session (SMD = −0.574, 95% CI = −1.025 to −0.124, *p* = 0.012, I^2^ = 67.6%). A stable and significant intervention effect was observed in the moderate-intensity group (3 to <6 METs; SMD = −0.377, 95% CI = −0.584 to −0.171, *p* < 0.001, I^2^ = 0.0%). In contrast, the low-intensity group (≤3 METs) exhibited the largest effect size (SMD = −0.704), but this finding was accompanied by a wider confidence interval (−1.183 to −0.225) and substantial heterogeneity (I^2^ = 53.9%).

**Table 4:** Pooled effect sizes and heterogeneity statistics from subgroup analyses of urban green exercise interventions on mental health.

Group	Sub-group	K	N	SMD	95% CI	*p*	I^2^
Duration(weeks)	<12	7	400	−0.481	−0.788 to −0.173	0.002	54.0%
	12 ≤ 16	5	455	−0.414	−0.656 to −0.172	0.001	37.0%
	>16	3	224	−0.261	−0.525 to −0.002	0.052	0.0%
Frequency(week)	1	8	633	−0.382	−0.746 to −0.018	0.001	58.2%
	2	6	328	−0.412	−0.638 to −0.187	0.000	0.0%
	≥3	1	118	−0.428	−0.687 to −0.169	0.040	0.0%
Time(min)	<20	5	579	−0.574	−1.025 to −0.124	0.012	67.6%
	20≤60	6	313	−0.387	−0.584 to −0.191	0.000	26.7%
	>60	4	187	−0.314	−0.605 to −0.023	0.034	0.0%
Interventions	<3	4	208	−0.704	−1.183 to −0.225	0.004	53.9%
	3 ≤ 6	6	373	−0.377	−0.584 to −0.171	0.000	0.0%
	>6	5	498	−0.287	−0.488 to −0.087	0.005	18.1%
Gender	Female<50	5	343	−0.331	−0.558 to −0.103	0.004	6.8%
	Female≥50	10	736	−0.453	−0.665 to −0.241	0.000	45.5%

K, number of trials; N, number of participants; SMD, standardized mean diference; CI, confidence interval; P, significant.

## 4 Discussion

A comprehensive synthesis of the clinical evidence on urban green exercise interventions for mental health was provided through a systematic review and meta-analysis, with the aim of elucidating potential underlying mechanisms and proposing scientifically effective green exercise protocols. A statistically significant positive effect of urban green exercise on the mental health of urban residents was observed (SMD = −0.40; 95% CI = −0.56 to −0.25; *p* < 0.001). The low heterogeneity between studies (I^2^ = 33.9%) suggests that this positive effect is consistent and robust across diverse research contexts. A dual-intervention approach integrating exposure to natural environments with physical activity is thereby established, providing strong empirical support for addressing the growing urban mental health crisis and offering novel perspectives for urban planning and public health policy.

In recent years, a substantial body of evidence from systematic reviews and meta-analyses has consistently confirmed the beneficial effects of green exercise on mental health and emotional regulation (15, 52, 53). For example, Huang et al. examined short-term, acute simulated green exercise interventions and demonstrated that physical activity performed in virtual or simulated natural environments can yield psychological restoration and mood enhancement (54). Wicks et al. compared outdoor physical activity in natural versus urban environments and found stronger immediate psychological benefits in natural settings; however, in that literature, “urban environments” typically refer to gray, sparsely vegetated streets rather than true urban green spaces (55). Notably, prior reviews have commonly adopted a broad definition of “green exercise,” combining remote wilderness areas and urban parks, which supports the establishment of overall benefits but may obscure differences between setting types (56, 57). A recent meta-analysis further suggests that exercise conducted in wild environments may produce greater mood-related benefits than in urban greenspaces, while also noting that the greater management and accessibility of urban greenspaces may confer unique health-promotion potential (28). Against this backdrop, the present study limits the geographic scope to urban green spaces and systematically examines and confirms the beneficial impact of urban green exercise on mental health. Specifically, compared with indoor exercise, urban green exercise not only improves mental health through the physiological pathways inherent to physical activity (e.g., endorphin release, cardiovascular adaptations) (58) but also leverages the passive restoration properties of natural environments to generate synergistic, more-than-additive effects. This combination aligns with the core propositions of restoration and stress-reduction frameworks, indicating that even fragmented, managed “doses” of urban nature, when coupled with physical activity, can elicit substantial psychological restoration. Moreover, relative to exercise in forested settings, activity in city parks and community greenways aligns more closely with residents’ daily routines, reduces participation barriers, and represents a sustainable, low-cost public health solution—consistent with the “urban nature therapeutics” perspective (59) and with large-scale, population-based evidence demonstrating significant mental health benefits from activities in urban parks (60).

To develop a scientific “green prescription, “it is necessary to address a more nuanced question: “What form of urban green exercise is most effective?” An in-depth subgroup analysis will now be conducted to examine the moderating roles of key intervention parameters, including duration, frequency, session length, gender composition, and METs. With regard to intervention duration, significantly larger effects on mental health were observed for short-term interventions (<12 weeks) compared to medium-term (12 to <16 weeks) and long-term (≥16 weeks) interventions. This finding is consistent with Attention Restoration Theory in environmental psychology and is supported by existing research, further validating the effectiveness of short-term interventions in promoting mental health recovery (61, 62). However, from a theoretical perspective on exercise interventions, this finding appears somewhat paradoxical, as continuous, long-term regular exercise would generally be expected to yield more stable and enduring mental health improvements. Several plausible mechanisms could account for this apparent “short-term advantage.” First, the initial phase of an intervention often elicits the greatest levels of participant enthusiasm and adherence, primarily driven by novelty and initial motivation (63, 64). Over time, longer-term interventions may encounter challenges such as participant fatigue or declining interest, potentially resulting in a diminished “effective dose” of the intervention. Second, a significant limitation in the current body of literature is the prevalent lack of long-term follow-up data, which may result in the underestimation of delayed or sustained effects of longer-term interventions because the analysis is primarily based on immediate post-intervention outcomes. In terms of intervention frequency, the largest effect size was observed for interventions conducted three or more times per week (SMD = −0.428; 95% CI = −0.687 to −0.169), while the effects of once- and twice-weekly interventions (SMD = −0.382 and −0.412, respectively) were relatively smaller. This result is consistent with the “dose–response” relationship described in exercise psychology (65). It has been suggested that higher frequency exercise may enhance mental health benefits by continually activating stress-buffering systems and reducing cortisol levels (66). Notably, the heterogeneity for the twice-weekly group was 0%, suggesting that this frequency may represent an optimal balance between efficacy and feasibility and may therefore be suitable for standardized intervention recommendations. With regard to session duration, interventions lasting <20 min and 20 ≤ 60 min were found to demonstrate similar and superior effects in improving mental health compared to those lasting over 60 min, a result consistent with existing empirical evidence (67). It has been suggested that short-duration green exercise may also yield additional mental health benefits by optimizing time efficiency and enhancing self-efficacy and a sense of accomplishment (68). Furthermore, it has been demonstrated that moderate-duration urban green exercise can significantly increase the activity of gamma-aminobutyric acid (GABA) in the central nervous system, thereby providing a neurochemical basis for emotional stability (69). In terms of intervention intensity, the largest mental health effect was observed for low-intensity interventions (3 ≤ METs), followed by moderate-intensity (3 < 6 METs), while higher-intensity exercise demonstrated the smallest effect. This pattern may reflect an “inverted U-shaped” relationship between exercise intensity and mental health, in which low-to-moderate intensity exercise is most beneficial for mood enhancement, while high-intensity exercise may inhibit positive emotions due to increased physiological stress and fatigue. More pronounced mental health benefits from urban green exercise were observed among female participants, consistent with current research (70). From a neuroimaging perspective, more significant activation in brain regions associated with emotion regulation (e.g., the prefrontal cortex) during nature exposure has been observed in women, providing a biological basis for greater mental health gains from green exercise.

## 5 Limitations of the study

Although this meta-analysis systematically synthesized data from 15 randomized controlled trials and quantified the mental health benefits of urban green exercise, several limitations must be acknowledged.

First, the majority of included studies focused on short-term interventions, with follow-up durations typically not exceeding 3 months. The lack of medium- and long-term data limits our ability to evaluate the sustainability of urban green exercise benefits or identify potential rebound effects following intervention cessation.

Second, significant heterogeneity was observed in the types and modalities of physical activities employed (e.g., walking, cycling, aerobic training), despite efforts to standardize exercise intensity using METs. These intervention differences may introduce variability in psychological outcomes due to differing levels of social interaction, environmental immersion, or activity familiarity, potentially biasing effect estimates.

Third, many studies were characterized by small sample sizes and methodological shortcomings, such as inadequate reporting of randomization procedures, lack of allocation concealment, and limited implementation of blinding protocols. These factors contributed to an elevated risk of bias and increased the likelihood of small-study effects and publication bias. Therefore, enhancing methodological rigor should be prioritized in future investigations.

To improve the external validity and causal explanatory power of future research, it is recommended that multi-center, large-sample RCTs be conducted with standardized intervention designs and extended follow-up periods. Such efforts will be crucial for developing robust, evidence-based protocols for the integration of urban green exercise into mental health promotion and public health planning.

## 6 Conclusion

This meta-analysis demonstrated a moderate yet statistically significant positive effect of urban green exercise on the mental health of adults. Interventions implemented in city parks, green spaces, and urban forests were most effective when designed as low-intensity activities (≤3 METs), conducted once or twice weekly, for sessions lasting 20–60 min, over a period shorter than 12 weeks. Notably, interventions featuring three or more sessions per week, each lasting ≤20 min, also yielded significant mental health improvements. In contrast, high-intensity or long-duration interventions (>16 weeks) were less effective. Female participants appeared to derive more substantial psychological benefits from urban green exercise. The results of subgroup analyses confirmed that key variables—such as intervention frequency, duration, and intensity—significantly moderated the effectiveness of urban green exercise. These findings support the establishment of evidence-based guidelines for the design and implementation of urban green exercise programs as a mental health promotion strategy. However, the generalizability of these findings is constrained by the limited number of included studies, small sample sizes, and varying methodological quality. Therefore, further validation through high-quality, large-scale RCTs is needed to reinforce the robustness and applicability of these conclusions.
